# MiRNA-29c regulates the expression of inflammatory cytokines in diabetic nephropathy by targeting tristetraprolin

**DOI:** 10.1038/s41598-017-01027-5

**Published:** 2017-05-24

**Authors:** Jia Guo, Jing Li, Jing Zhao, Shuguang Yang, Luyao Wang, Genyang Cheng, Dong Liu, Jing Xiao, Zhangsuo Liu, Zhanzheng Zhao

**Affiliations:** 1 Nephrology Hospital, The First Affiliated Hospital of Zhengzhou University, NO. 1 Jianshe Eastern Road, 6th Floor of NO. 7 Building, Erqi District, Zhengzhou 450052 China; 2 Zhengzhou University Institute of Nephrology, Zhengzhou, 450052 China; 3 Institute of Clinical Medicine, The First Affiliated Hospital of Zhengzhou University, Zhengzhou, 450052 China

## Abstract

Diabetic nephropathy is one of the most prevalent chronic complications of Diabetes mellitus, but its pathogenesis remains elusive. This study was designed to determine the role of tristetraprolin (TTP), inflammatory cytokines and microRNAs (miRNAs) in DN. The blood and urine samples were obtained from 32 patients with DN, 33 patients with type 2 DM, and 35 normal healthy subjects as controls. Renal tissue samples were also obtained from 10 DN patients and 10 normal controls. The miRNA microarray analyses were performed in pooled plasma and urine sediment samples of eight DN patients and eight age- and sex-matched health control subjects and three paired renal tissues from patients with DN and normal controls. Conditionally immortalized mouse podocytes (MPC5) were used a cell model. The expressions of TTP and cytokines in patient samples and cultured cells were determined by qRT-PCR and Western blotting or ELISA. Our results indicated that miRNA-29c directly targeted TTP and promoted inflammatory response under hyperglycemic conditions. Overexpression of miRNA-29c in podocytes resulted in an increase in inflammatory cytokines and inhibition of miRNA-29c by using its inhibitor reduced the inflammatory cytokines in podocytes. Finally, miRNA-29c promoted the progression of DN by targeting TTP, providing a target for a therapeutic intervention of DN.

## Introduction

Diabetes mellitus (DM) is a chronic metabolic disease that is expected to be one of the leading causes of death world-wide in about two decades^1^. In 2012, the estimated global prevalence of DM was 8.3%, affecting more than 371 million adults worldwide^2^. By the end of 2030, its global prevalence is expected to rise by 55% with more than 592 million adults having DM^3^. Diabetic nephropathy (DN) is one of the most prevalent chronic complications of DM, occurring in one-third of diabetic patients, irrespective of the type of diabetes^4^.

The pathogenesis of DN has not been fully understood, but several factors may be involved, including hyperglycemia, advanced glycation end products, protein kinase C, oxidative stress, and poly (ADP-ribose) polymerase activation^5^. There is increasing evidence supporting that both activated innate immunity and inflammation are engaged in the DN pathogenesis^6^. The accumulation of inflammatory cells in the kidney is a key player in the induction of DN^7^ and blocking the recruitment of inflammatory cells to the kidneys prevents renal injury in animal models of DN^8^. Pro-inflammatory cytokines produced by inflammatory cells, such as interleukin (IL)-1, IL-6, IL-18, and tumor necrosis factor (TNF)-α, can directly damage kidney architecture, playing a pivotal role in the pathogenesis of DN^9^. Additionally, the elevated serum and urine levels of pro-inflammatory cytokines correlate with the progression of DN^10^.

However, the underlying mechanisms for inflammatory response in DN pathogenesis remain elusive. As a critical anti-inflammatory protein, TTP enhances the decay of mRNAs, conferring mRNA instability and degradation by binding to the conserved adenosine/uridine-rich element (ARE) present within the 3′-untranslated region (UTR) of mRNA transcripts of cytokines, such as IL-6 and TNF-α^11–14^. The role for TTP as an anti-inflammatory protein was first elucidated when the TTP knockout mouse developed a pro-inflammatory phenotype due to overexpression of TNF-α in macrophages, resulting in cachexia, myeloid hyperplasia, and a host of other inflammatory responses^15^. It has been shown that diabetic patients with clinical proteinuria are accompanied by decreased urinary and serum levels of TTP and increased levels of IL-6 and IL-18, and that decreased TTP expression might occur prior to the increase in IL-6 and IL-18^16^, suggesting that TTP is involved in the inflammatory response in DN and can be developed as a marker for diabetic kidney damage^16^.

More recently, the role of microRNAs (miRNAs) in regulation of gene expression and in the development and progression of various diseases, including DM, has been found; miRNAs regulate gene expression by base-pairing to partially complementary sites in the 3′-UTR of specific target mRNAs^17^. Emerging evidence suggests that miRNAs can be developed as important therapeutic approaches in a wide range of human diseases^17, 18^. Recent studies have also revealed the involvement of miRNAs in inflammation of DN^19–22^, indicating a rationale for developing miRNA therapeutics to treat DN.

The present study was designed to investigate the effects of miRNA-29c on the regulation of TTP and the expression of pro-inflammatory cytokines in patients with DN. The reasons for choosing miRNA-29c in the present study are as follows. Our previous studies have identified the relationship between TTP and DN patients with proteinuria^23^. Our previous microarray results also showed different expression levels of miRNA-29c in plasma, urinary sediment and renal tissues in patients with DN. Research findings from Chien *et al*. showed that miRNA-29a/b/c could reflect DN pathogenesis and serve as biomarkers during DN progression^24^.

Furthermore, research findings from Peng *et al*. have indicated that miR-NA-29a/c may have the potential to serve as alternative biomarker for DN and atherosclerosis in type 2 diabetes^25^. Thus, we generated hypothesis based on our previous findings and other reported studies.

## Results

### Characteristics of Study Subjects

65 diabetes patients and 35 normal control subjects were enrolled in the present study. The patients’ characteristics and the results of biochemical urine assays are shown in Table 1. There were no significant differences in age and gender among the three groups (*P* > 0.05).

**Table 1:** Characteristics of Patients and Controls and Laboratory Test Results.

	Control	T2DM Patients	DN Patients
n	35	33	32
Age (yr)	45.33 ± 4.12	55.9 ± 2.96	52.4 ± 4.53
BMI (kg/m2)	22.26 ± 0.44	25.57 ± 1.64*	24.1 ± 1.70
Course (yr)	—	4.4 ± 0.65	11.9 ± 2.02^#^
SBP (mmHg)	133.3 ± 2.31	125.8 ± 3.71	143.2 ± 4.42^#^
TG (mmol/L)	1.28 ± 0.20	1.51 ± 018	2.52 ± 0.41*^#^
TC (mmol/L)	3.20 ± 0.40	4.38 ± 0.37	4.48 ± 0.45*
UA (umol/L)	211.76 ± 21.65	276.67 ± 28.94*	318.12 ± 32.17*^#^
BUN (mmol/L)	5.06 ± 0.63	5.15 ± 0.46	11.65 ± 2.17*^#^
Cr (umol/L)	62.17 ± 9.73	57.20 ± 8.69	209.63 ± 58.06*^#^
HbA1c (%)	5.25 ± 0.16	9.10 ± 0.44**	8.01 ± 0.47**
UAER (ug/min)	8.01 ± 2.54	14.18 ± 2.87	601.42 ± 133.29*^#^

T2DM: type 2 diabetes mellitus, DN: diabetic nephropathy, BMI: body mass index, SBP: systolic blood pressure, TG, triglyceride, TC: serum total cholesterol, UA: uric acid, BUN: blood urea nitrogen, Cr: serum creatinine, HbA1c: glycated hemoglobin, UAER: urinary albumin excretion rates.

*Compared to the control group: *P* < 0.05.

**Compared to the control group: *P* < 0.01.

^#^Compared to the T2DM group: *P* < 0.05.

### There are Significant Differences in miRNA Expressions between the DN Patients and Controls

According to miRNA Array analysis, the miRNAs with more than 2-fold changes between the two groups were identified. For the kidney tissues, there were 152 miRNAs with increased levels and 167 miRNAs with decreased levels in the DN group, compared with the control group. For the plasma samples, the DN group showed 103 miRNAs with increased levels and 200 miRNAs with decreased levels. For urinary sediment samples, the DN group showed 770 miRNAs with increased levels and 239 miRNAs with decreased levels.

### There are Significant Differences in the miRNA-29c Levels in Plasma, Urine and Renal Tissue Samples between DN Patients and Controls

Compared with the control and T2DM groups, the plasma miRNA-29c expression level in DN group was increased, but its levels in urinary sediment and renal tissues were decreased. Compared with the NC group, the plasma miRNA-29c level in T2DM group was increased, but its levels in the urine sediment and kidney tissues were decreased.

### There are Significant Differences in Plasma Levels of TNF-α and IL-6 and Urine Levels of TNF-α and IL-6 between DN Patients and Controls

As shown in Table 2, the plasma levels of IL-6 in the NC and T2DM groups were significantly lower than that in the DN group (*P* < 0.05). The plasma TNF-α levels in the NC and T2DM groups were also significantly lower than that in the DN group (*P* < 0.05). Similarly, the IL-6 and TNF-α levels in the urine samples from the NC and T2DM groups were significantly lower than that from the DN group, respectively (*P* < 0.05), which was consistent with the aforementioned results of the plasma samples.

**Table 2:** The Levels of IL-6, TNF-α and TTP in Plasma and Urine Samples.

	Control subjects	T2DM patients	DN patients
Plasma			
IL-6 (pg/ml)	3.685 ± 0.471	4.159 ± 0.443	7.397 ± 2.196*^#^
TNF-α (pg/ml)	2.010 ± 0.284	2.373 ± 0.132	8.087 ± 3.122*^#^
TTP (pg/ml)	284.19 ± 20.61	196.14 ± 17.11*	81.34 ± 16.51*^#^
Urine			
IL-6 (pg/ml)	2.373 ± 0.585	4.242 ± 0.668	6.176 ± 0.664*^#^
TNF-α (pg/ml)	7.202 ± 1.42	11.241 ± 1.53	22.639 ± 1.47*^#^
TTP (pg/ml)	244.19 ± 18.37	160.03 ± 19.41*	68.61 ± 17.49*^#^

*Compared to control subjects, *P* < 0.05.

^#^Compared to T2DM patients, *P* < 0.05.

### There are Correlations between miRNA-29c Levels and Cytokine Levels

**Figure 1:**
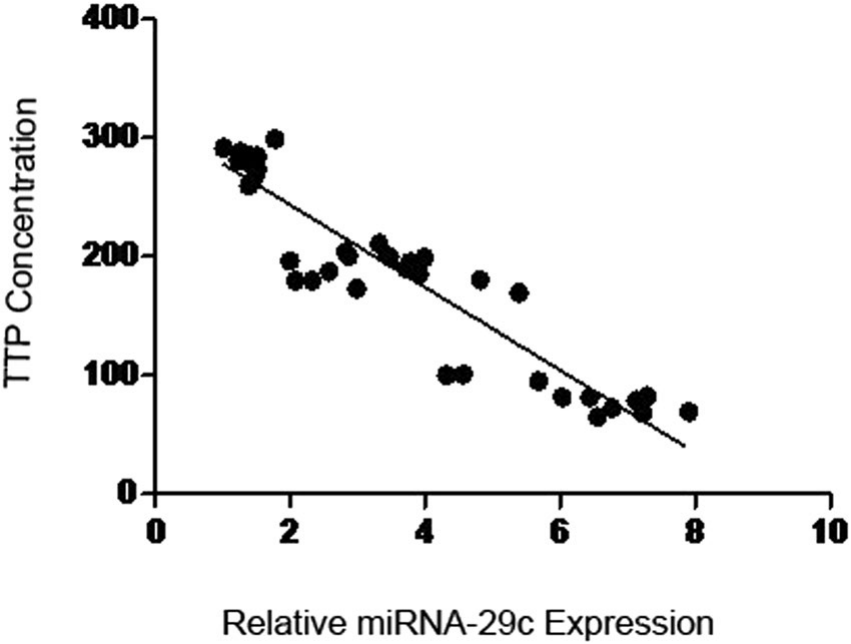
Correlation analysis between miRNA-29c and TTP.

**Figure 2:**
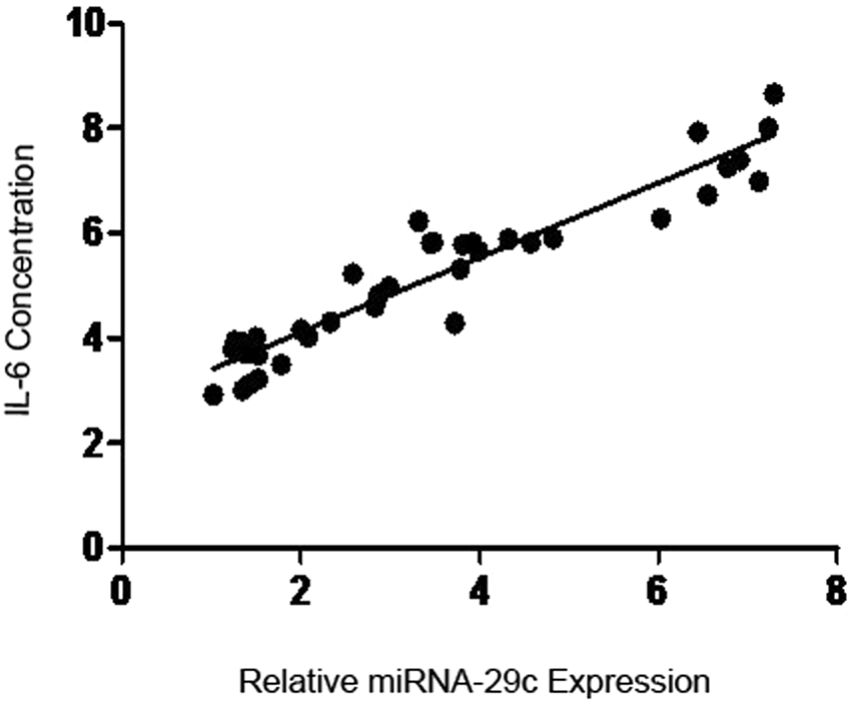
Correlations between miRNA-29c and IL-6.

Our results showed that, in patients with DN, the miRNA-29c levels were positively correlated with the levels of IL-6 (correlation coefficient r = 0.879, *P* < 0.05, Figs 1 and 2). The miRNA-29c levels were negatively correlated with the levels of TTP, (r = −0.9, *P* < 0.05). The miRNA-29c levels were positively correlated with the levels of IL-6 (r = 0.879, *P* < 0.05). Additionally, the miRNA-1207-5p levels were positively correlated with the levels of TTP (r = 0.8, *P* < 0.05), but negatively correlated with the levels of IL-6 (r = −0.818, *P* < 0.05).

### There are Correlations between miRNA-29c Levels and Clinical Parameters

**Figure 3:**
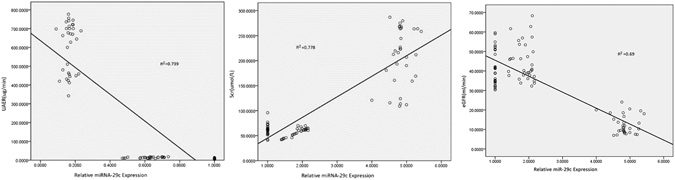
The Correlations between miRNA-29c Levels and Other Clinical Parameters. (**A**) The Correlations between miRNA-29c Levels and UAER. (**B**) The Correlations between miRNA-29c Levels and Scr. (**C**) The Correlations between miRNA-29c Levels and eGFR.

There was a negative correlation between miRNA-29c levels and UAER (Uri-nary Albumin Excretion Rates), as UAER trends to increase, miRNA-29c levels trend to decrease (Fig. 3A). There was a positive correlation between miRNA-29c levels and Scr, as Scr trends to increase, miRNA-29c levels trend to increase (Fig. 3B). There was a negative correlation between miRNA-29c levels and eGFR, as eGFR trends to increase, miRNA-29c levels trend to decrease (Fig. 3C).

### There are Up-regulation of miRNA-29c and Inflammatory Cytokines and Down-regulation of TTP in Podocytes under Hyperglycemic Conditions

**Figure 4:**
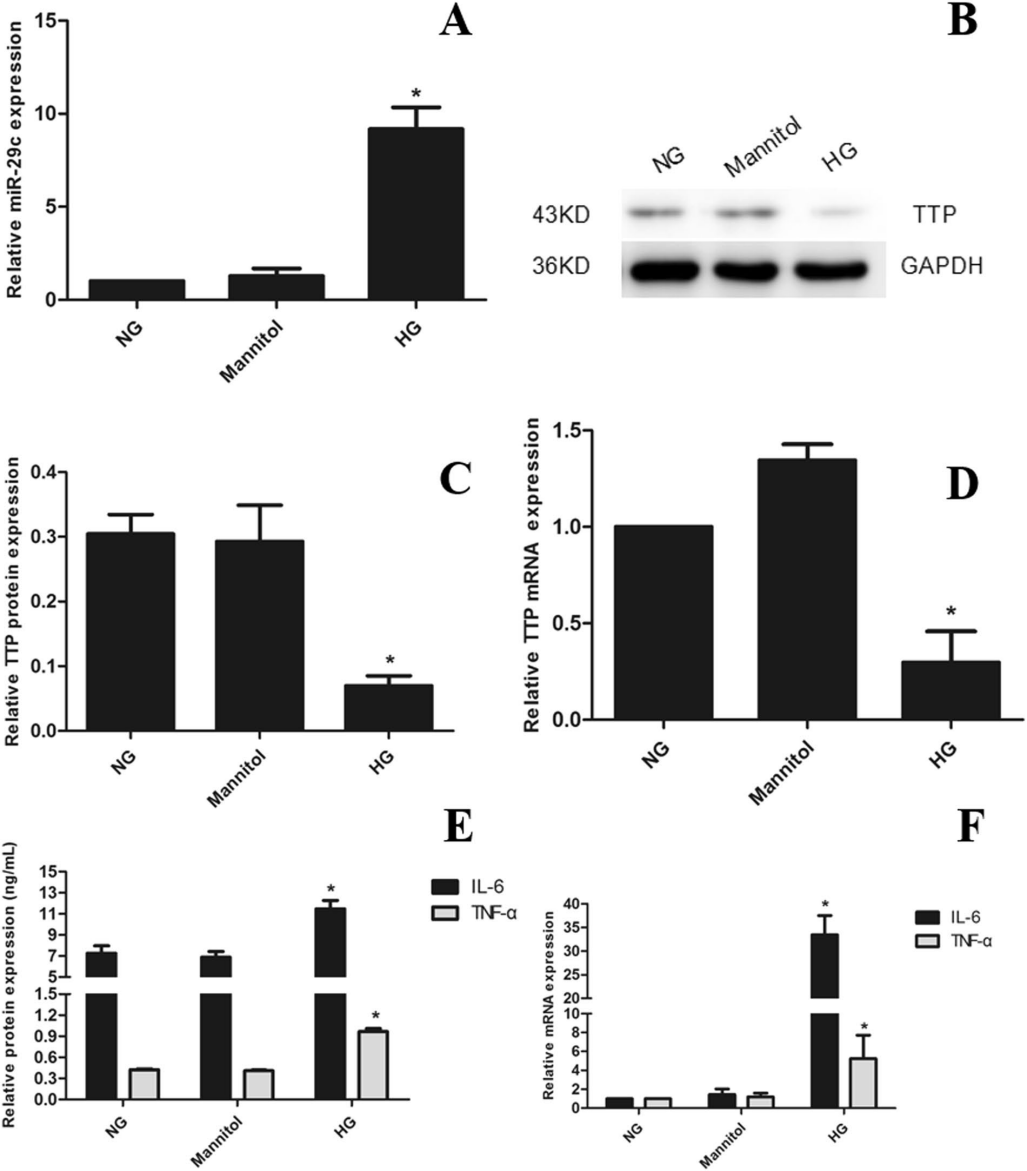
Up-regulation of miRNA-29c and inflammatory cytokines and down-regulation of TTP in podocytes. (**A**) Real-time qPCR analysis shows miRNA-29c expression in podocytes treated with high glucose (25 mM) for 6 h as com-pared with podocytes treated with either normal glucose (5.6 mM) or mannitol (25 mM). Measured transcript levels were normalized to U6 snRNA expression. (**B**) Podocytes were cultured in high glucose for 24 h as compared with normal glucose or mannitol, TTP protein expression was assessed by Western blot, GAPDH served as an internal control. (**C**) The quantified Western blot data shown in graph format. (**D**) qRT-PCR analysis of TTP mRNA expression in hyperglycemic condition. (**E**) IL-6 and TNF-α protein expression by ELISA analysis in hyperglycemic condition for 48 h. (**F**) qRT-PCR analysis of TTP mRNA expression in hyperglycemic condition. Results were obtained from three independent experiments. Data are shown as mean ± SEM (n = 3). *P < 0.05 compared with control.

As aforementioned, miRNA-29c was differentially expressed in blood plasma, urinary sediment, and kidney tissues of patients with DN, compared with the NC and DM controls. It has been reported the expression of miRNA-29c is differentially up-regulated in cultured podocytes and kidney microvascular endothelial cells under hyperglycemic conditions as well as in glomeruli from diabetic db/db mice with DN^22^. In the present study, we analyzed the expression of miRNA-29c in cultured podocytes at various time points (6, 12, and 24 h) following stimulation with high glucose (25 mM). The expression of miRNA-29c was significantly increased following 6-h exposure to high glucose, but not to mannitol (Fig. 4A). Western blotting analysis showed that the expression of TTP was down-regulated under high glucose condition for 24 h (Fig. 4B and C), consistent with the qRT-PCR results (Fig. 4D). Extending exposure time to 48 h, the expression levels of IL-6 and TNF-α were higher under hyperglycemic conditions, compared with that under normal and mannitol conditions (Fig. 4E). Similarly, the real-time qPCR analysis indicated that high glucose treatment led to an increase in IL-6 and TNF-α expression (Fig. 4F). Taken together, these findings demonstrated that miRNA-29c and inflammation cytokines were up-regulated in response to hyperglycemic conditions whereas TTP was down-regulated. Alterations of miRNA-29c and TTP expression were prior to that of inflammation cytokines, indicating that miRNA-29c and TTP were involved in the inflammatory response under hyperglycemic conditions.

### TTP is a Molecular Target of miRNA-29c

**Figure 5:**
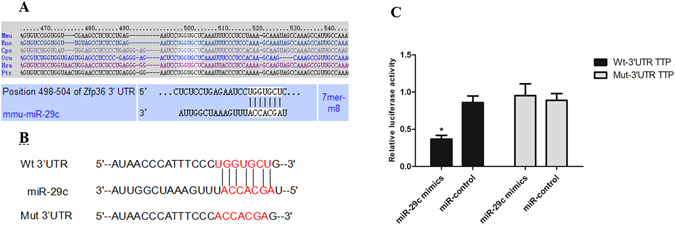
miRNA-29c downregulates TTP by interacting with its 3′-UTR. (**A**) A predicted miRNA-29c target site resides at nucleotides 498–504 (shown in the white box) of the mouse TTP 3′-UTR and is highly conserved in several species. (**B**) The wild-type and mutant sequences of TTP 3′-UTR. (**C**) Differentiated podocytes were co-transfected with Wt or Mut TTP 3′-UTR-pmirGLO and miRNA-29c mimics or miR-control. After 36 h, cells were lysed and luciferase activities were detected by Dual-Luciferase Reporter Assay Kit. Luciferase activities were normalized to Renil-laluciferase. Results were obtained from three independent experiments. Data are shown as mean ± SEM. *P < 0.05 compared with Mut TTP 3′-UTR and miRNA-29c mimics co-transfected group.

We next set out to identify the potential target(s) of miRNA-29c under hyperglycemic conditions. There were at least eight previously reported targets for the miRNA-29c, including collagen, tumor necrosis factor alpha-induced protein 3 (TNFAIP3), sprouty homolog 1 (Spry1), T cell lymphoma invasion and metastasis 1 (TIAM1), matrix metalloproteinase 2 (MMP2), integrin β1, DNA methyltransferase 3 (DNMT3), and IGF-1^22, 26–31^. Using TargetScan program, we identified TTP as a new target of miRNA-29c. We found a highly conserved binding site for miRNA-29c in the 3′-UTR region of TTP in several species (Fig. 5A). The alignment between miRNA-29c and 3′-UTR of mouse TTP is shown in Fig. 5A. The 3′-UTR of the mouse TTP gene contains a 7-mer (UGGUGCU), which is complementary to the seed region of miRNA-29c. Thus, we hypothesized that TTP might serve as a target for miRNA-29c in DM.

To address whether binding of miRNA-29c to TTP mRNA leads to its translational suppression, we cloned 3′-UTR of the mouse TTP gene into luciferase reporter vector pmirGLO. We also generated a mutant where the putative miRNA-29c binding site UGGUGCU was mutated into ACCACGA (Fig. 5B). Transient co-transfection of miRNA-29c mimics with Wt TTP 3′-UTR resulted in a significant repression of luciferase reporter gene in podocytes, whereas transfection of cells with miR-control did not have any effect on the expression of luciferase (Fig. 5C). However, mutations within the binding site of TTP abrogated the effect of miR-NA-29c mimics (Fig. 5C). Taken together, these results suggested that miRNA-29c directly targeted and inhibited TTP by binding to the 3′-UTR binding site of TTP.

**Figure 6:**
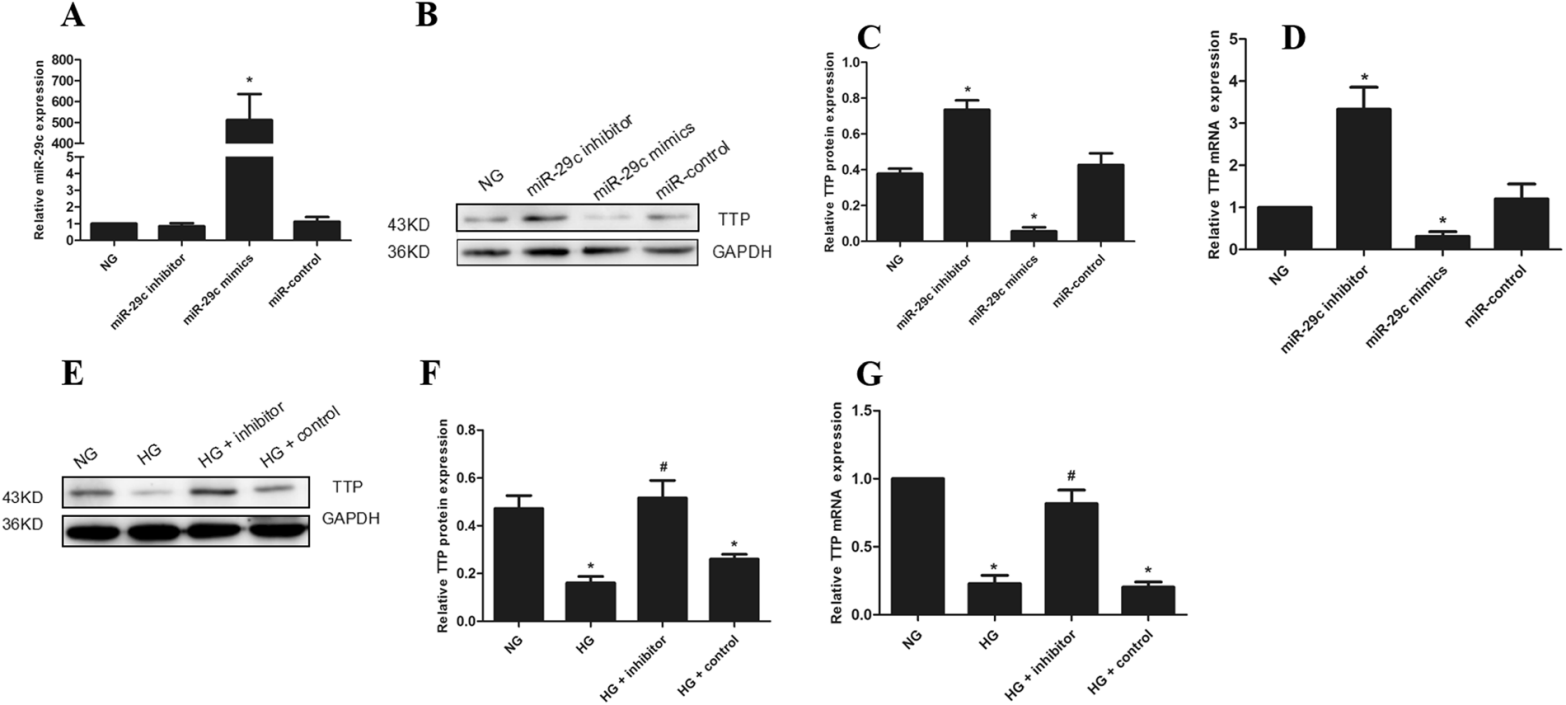
miRNA-29c targets TTP. (**A**) The expression of miRNA-29c in podocytes transfected with mimics (30 nM) or inhibitors (100 nM) for 6 h. (**B**) Western blot analysis showed the expression of TTP in podocytes transfected with mimics or inhibitors for 36 h. (**C**) The quantified Western blot data shown in graph format. (**D**) qRT-PCR analysis of TTP mRNA expression in podocytes transfected with mimics or inhibitors. (**E**) Podocytes were transfected with miRNA-29c inhibitor (100 nM) and treated with HG (25 mM) for 36 h as compared with NG. (**F**) The quantified Western blot data shown in graph format. (**G**) qRT-PCR analysis of TTP mRNA expression in podocytes transfected with miRNA-29c inhibitors (100 nM) and treated with HG (25 mM) for 24 h. Results were obtained from three independent experiments. The data are shown as mean ± SEM. *P < 0.05 compared with control. ^#^ *P* < 0.05 compared with high glucose.

To further provide evidence for that TTP is a target of miRNA-29c, we transfected podocytes with miRNA-29c mimic or inhibitor. Using qRT-PCR analysis, we confirmed that the expression of miRNA-29c with miRNA-29c mimic transfection was significantly higher than that with miRNA-29c inhibitor and miR-control transfection (Fig. 6A). The results indicated that miRNA-29c inhibitors had no effect on the miRNA-29c expression, only inhibiting the function of miRNA-29c. Using Western blot analysis, we addressed whether miRNA-29c overexpression had an inhibitory effect on endogenous TTP protein expression. As shown in Fig. 6B and C, the increased levels of TTP were found in the inhibitors transfected podocyte, whereas reduced levels of TTP in the mimic transfected podocyte, compared with the control podocyte. Similarly, real-time qPCR analysis demonstrated the similar results (Fig. 6D). These findings suggested that miRNA-29c down-regulated TTP expression in podocytes.

To further examine the effect of miRNA-29c on TTP, we transfected podocytes with miR-inhibitors and then treated podocytes with high glucose (25 mM). Western blot and qRT-PCR analysis confirmed that high glucose-induced down-regulation of TTP was reversed when podocytes were co-transfected with a miRNA-29c inhibitor (Fig. 6E,F and G). These findings indicated that high glucose down-regulated TTP expression through a miRNA-29c-regulated pathway, further confirming that TTP is a direct target of miRNA-29c.

### miRNA-29c Modulates Inflammatory Cytokines by Targeting TTP

**Figure 7:**
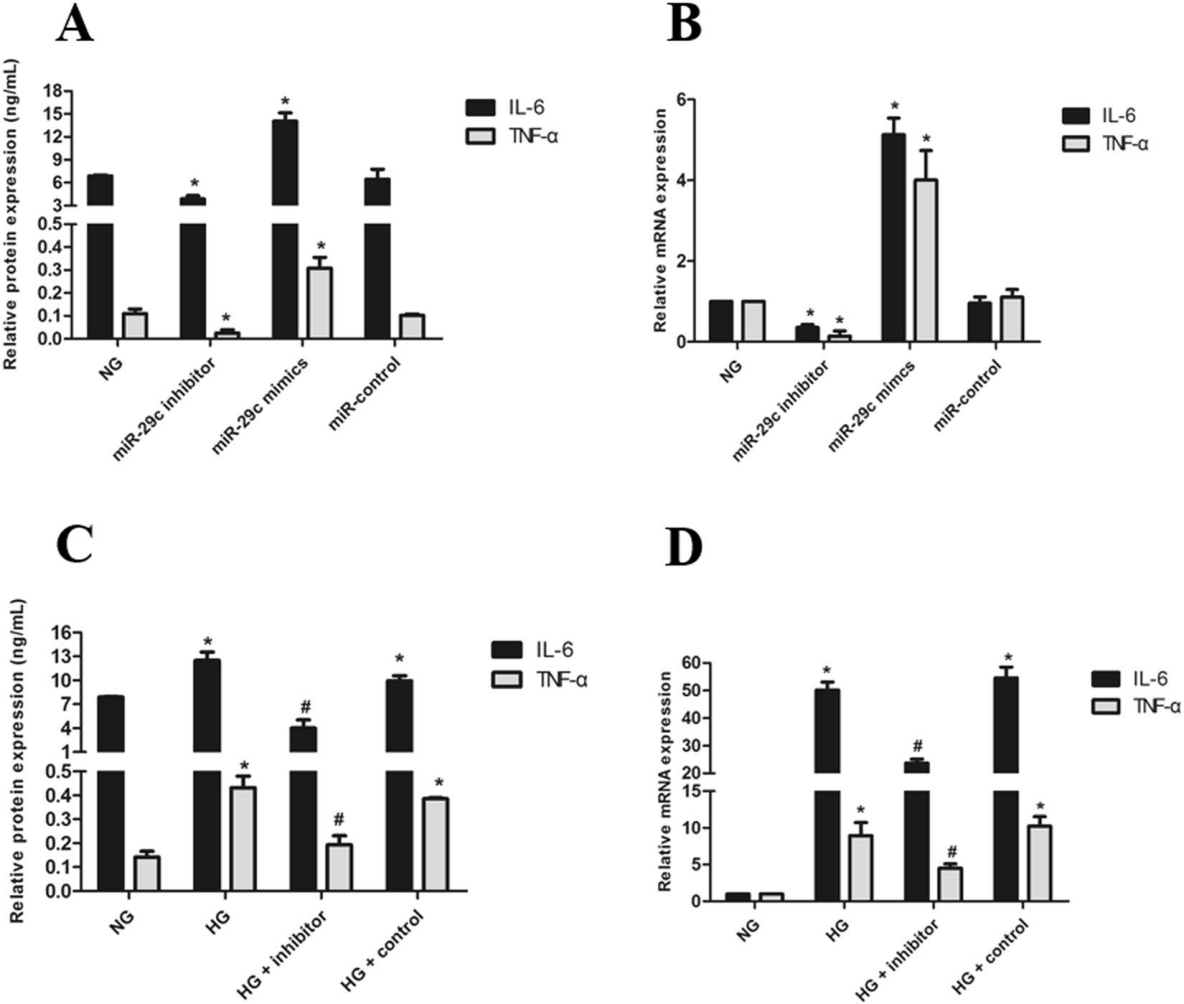
miRNA-29c promotes inflammatory cytokines synthesis. (**A**) ELISA analysis showed the expression of inflammatory cytokines in podocytes transfected with mimics (30 nM) or inhibitors (100 nM) for 48 h. (**B**) qRT-PCR analysis of in-flammatory cytokines expression in podocytes transfected with mimics or inhibitors. (**C**) ELISA analysis showed the expression of inflammatory cytokines in podocytes were transfected with miRNA-29c inhibitor (100 nM) and treated with HG (25 mM) for 48 h as compared with NG. (**D**) qRT-PCR analysis of inflammatory cytokines mRNA expression in podocytes transfected with miRNA-29c inhibitors (100 nM) and treated with HG (25 mM) for 24 h. Results were obtained from three independent experiments. Data are shown as mean ± SEM. *P < 0.05 compared with control. ^#^ *P* < 0.05 compared with high glucose group.

We next examined whether miRNA-29c might be an active component of the inflammatory response in DN. We transfected miRNA-29c mimics or inhibitors into the podocytes. Western blot and qRT-PCR analyses demonstrated that the raised miRNA-29c expression in podocytes resulted in an increase in inflammatory cytokines. In contrast, loss-of-function of miRNA-29c by using its inhibitor led to reductions in inflammatory cytokines in podocytes (Fig. 7A and B). We transfected podocytes with a miRNA-29c inhibitor, and then treated podocytes with high glucose (25 mM). Western blot and qRT-PCR analyses confirmed that high glucose-induced up-regulation of inflammatory cytokines was reversed when podocytes were co-transfected with a miRNA-29c inhibitor (Fig. 7C and D), indicating a potent pro-inflammatory effect of miRNA-29c in podocytes.

### Renal Tubular Epithelial Cells Exposed to High Glucose Down-Regulate TTP

Our previous study used SD rats to investigate the effect of peritoneal dialysis (PD) with high-glucose dialysis fluid on the VEGF family, tristetraprolin (TTP), angiogenesis and lymphangiogenesis^32^. The study findings indicated the expression of tristetraprolin gradually decreased under hyperglycemic condition. Our experiments also found that the expression of tristetraprolin decreased in human renal tubular epithelial cells under hyperglycemic condition. Research findings from Khera *et al*.^33^ have indicated that inflammatory cells down-regulated tristetraprolin. Currently, only our research team has been investigating the effects of miRNA-29c regulating tristetraprolin.

**Figure 8:**
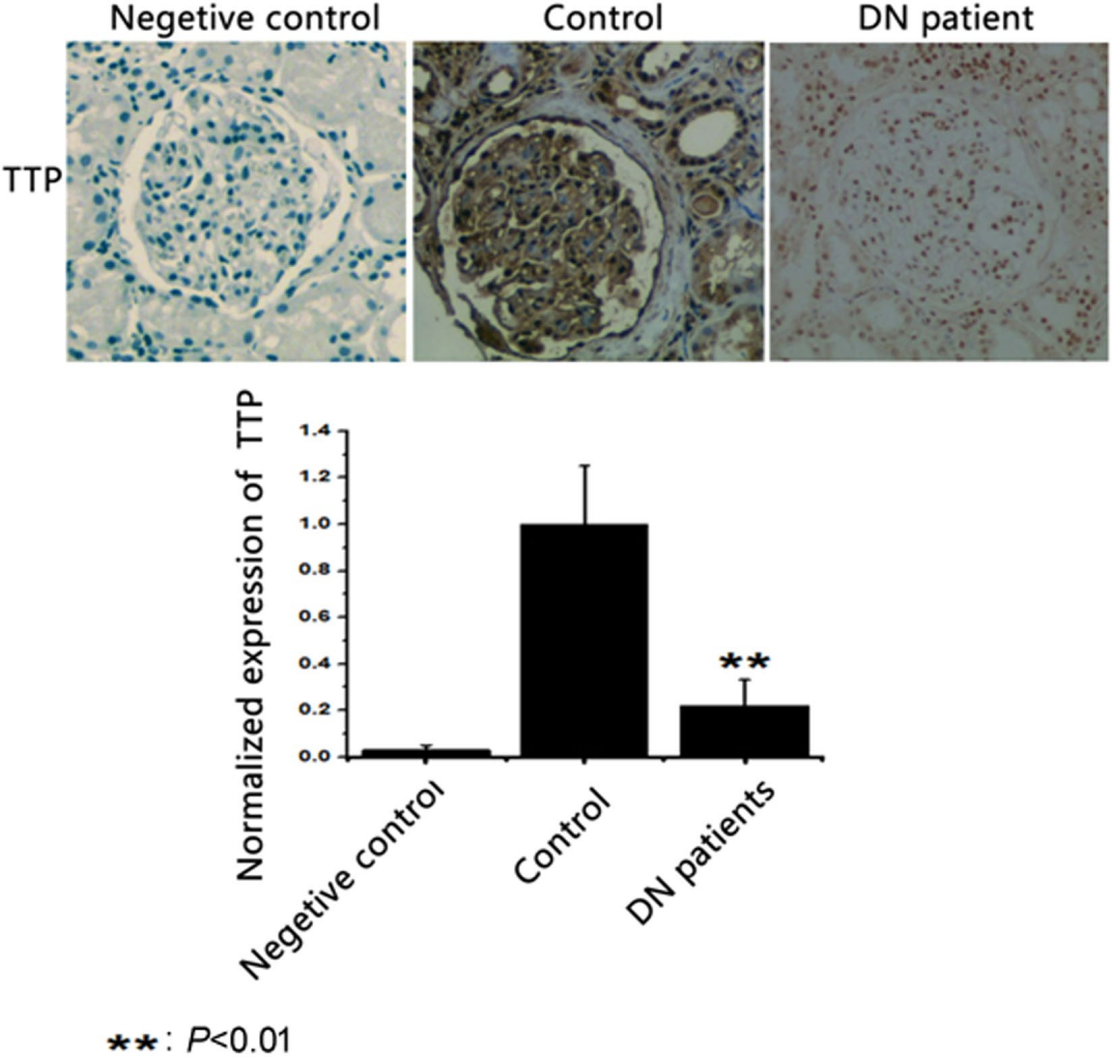
The Expression of TTP in Kidney Samples.

This study utilized immunohistochemistry to evaluate the expression of TTP in kidney samples. Three groups were established including negative control, control and DN patients. The negative control group used phosphate-buffered saline (PBS), the control group used normal renal tissue, and the DN patients group used the renal tissues of DN patients. Our findings indicated normalized expression of TTP for the negative control group was 0.03 ± 0.02, for the control group was 1.00 ± 0.25, and for the DN patients group was 0.22 ± 0.11. Compared to the control group, the expression of TTP for the DN patients group was significantly decreased, and the difference was statistically significant (*P* < 0.01) (Fig. 8).

**Figure 9:**
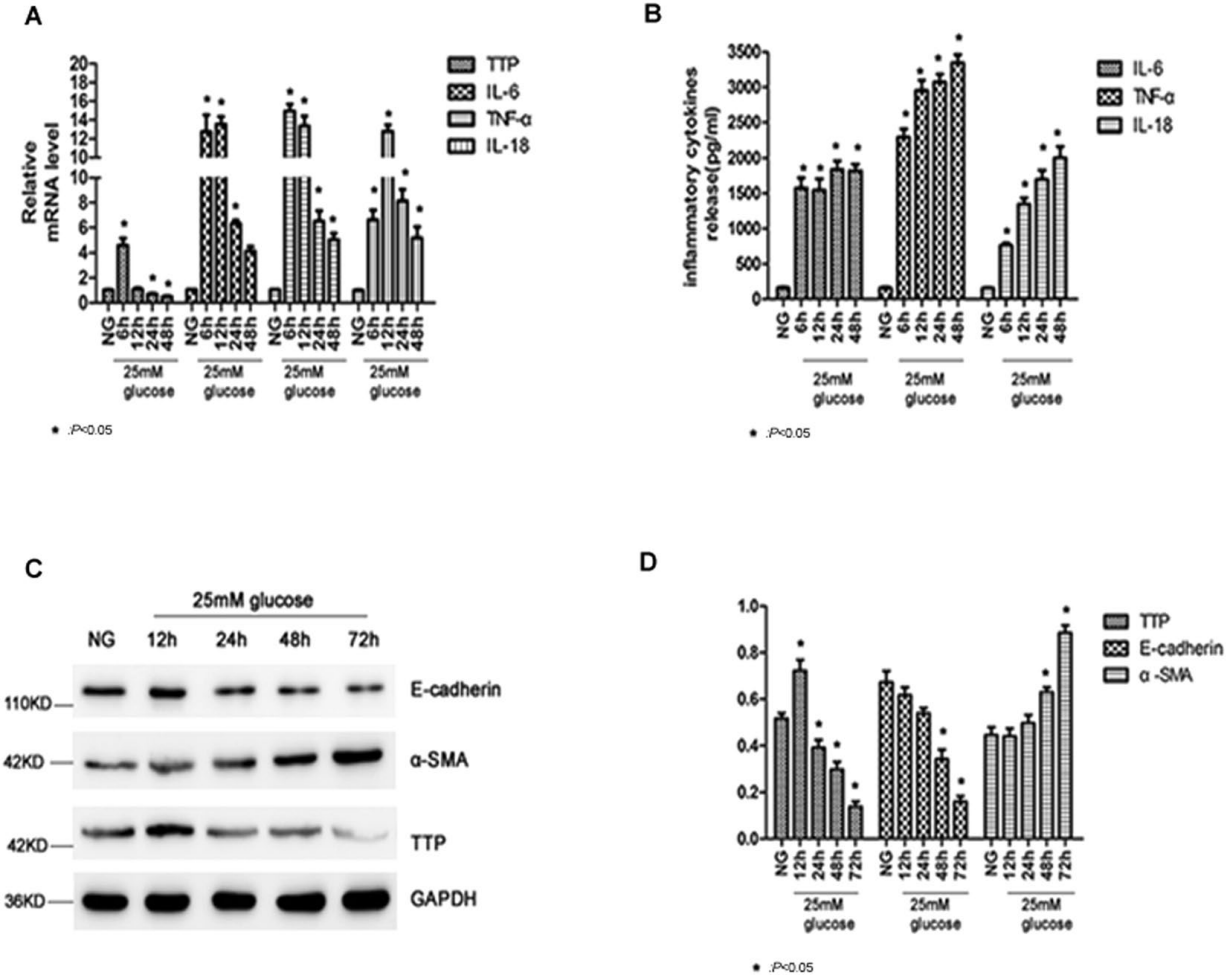
The Expressions of Renal Tubular Epithelial Cells Exposed to High Glucose. (**A**) The mRNA expressions of TTP, IL-6, TNF-α and IL-18. (**B**) The expression of inflammatory cytokines. (**C**) Western blot result regarding the expressions of TTP and transition-related proteins in renal tubular epithelial cells. (**D**) The expressions of TTP and transition-related proteins in renal tubular epithelial cells. *P < 0.05 compared with control.

We also evaluated the expressions of renal tubular epithelial cells exposed to high glucose. Figure 9A indicated the mRNA expressions of TTP, IL-6, TNF-α, and IL-18. Results showed high glucose up-regulated the expressions of inflammatory cells (IL-6, TNF-α, and IL-18), and down-regulated the expression of TTP. ELISA assay was used and results indicated high glucose up-regulated the expression of inflammatory cytokines (Fig. 9B). Western blot was used to detect the expressions of TTP and transition-related proteins of renal tubular epithelial cells. The results showed the high glucose down-regulated the expression of TTP in renal tubular epithelial cells, up-regulated the expression of marker protein α-smooth muscle actin (α-SMA) in fibroblast cells, and down-regulated marker protein E-cadherin in renal tubular epithelial cells (Fig. 9C). There was a statistical significant difference be-tween DN patients and control groups (*P* < 0.05) (Fig. 9D).

## Discussion

Our findings from the present study indicated that miRNA-29c directly targeted TTP and promoted inflammatory response under hyperglycemic conditions. Previous reports have suggested that miRNAs are important biomarkers of various complex diseases in humans^17, 18^, including DN^20–22^. To identify key regulatory miRNAs in DN, it would be necessary to examine miRNA expression patterns in an unbiased manner. Accordingly, we examined the expression profiles of miRNAs in samples of plasma, urinary sediments and kidney tissues from patients with T2DM, DN and control subjects. The results indicated that plasma miRNA-29c expression level increased, while miRNA-29c level in urinary sediment and renal tissues decreased differently in T2DM and DN patients. The finding of increasing plasma level was consistent with the results from Chen *et al*.^34^. However, the possible reasons for different miRNA-29c expression levels remain unclear. It could be related to pathophysiological response in hyperglycemic conditions, and further investigation may be required in the future.

We also demonstrated that up-regulation of miRNA-29c and inflammatory cytokines IL-6/TNF-α occurred under hyperglycemic conditions *in vitro*. Indeed, over-expression of miRNA-29c in podocytes by using its mimics resulted in an increase in inflammatory cytokines and inhibition of miRNA-29c by using its inhibitor reduced the inflammatory cytokines in podocytes. Our results indicated that miRNA-29c may serve as a promotive factor in the development and progression of DN, which was consistent with the results reported recently^22^. Interestingly, recent reports have also shown that miR-155 and/or miR-146a are involved in DN, through regulation of glomerular endothelial injury^20, 21^.

It is also important to emphasize that several recent studies have implicated the potential role of miRNAs as regulators of inflammatory response. For instance, the expression of miR-155 and miR-146a is increased in DN patients and miR-155 and miR-146a could significantly induce renal inflammation and contribute to glomerular endothelial injury under hyperglycemic conditions^21^. Serum miR-145 is significantly increased and positively correlated with plasma hs-CRP and serum IL-6 in patients with acute ischemic stroke^35^. Different from findings aforementioned, most studies have demonstrated an anti-fibrosis and nephroprotective role of miRNA-29 in the renal and cardiac fibrosis^36, 37^. Wang *et al*.^37^ have observed low levels of miRNA-29 family in three models of renal fibrosis: early diabetic renal fibrosis, advanced diabetic renal fibrosis, and advanced nondiabetic kidney disease. Furthermore, profibrotic cytokine TGF-β1 reduces the expression of the miR-NA-29a/b/c family, which targets collagen gene expression in proximal tubular epithelium cells, primary mesangial cells, and podocytes^38^. The miRNA-29 is a downstream inhibitor of TGF-β/Smad3 signaling pathway and negatively regulated by TGF-β/Smad3^39^. Overexpression of miRNA-29 prevents progressive renal fibrosis^32^.

Another major finding of our study was the identification of TTP as a novel target of miRNA-29c. TTP has been found to serve as an anti-inflammatory protein^10–13^. Liu *et al*. have shown that diabetes with clinical proteinuria is accompanied by decreased urinary and serum level of TTP and increased levels of IL-6 and IL-18, and decreased TTP expression might occur prior to the increase in IL-6 and IL-18^15^. In our study, TTP was down-regulated, whereas IL-6 and TNF-α were up-regulated under hyperglycemic conditions, and alteration of TTP expression was prior to that of IL-6 and TNF-α. It is becoming increasingly clear that most miRNAs are promiscuous and target multiple genes^40, 41^. The miRNA-29 family is a good example because they exert their effects through modulating multiple targets. Indeed, Collagen, TNFAIP3, Spry1, TIAM1, MMP2, integrin β1, DNMT3, IGF-1 have all been previously validated as direct targets of miRNA-29c^22, 26–31^. Our findings demonstrated that TTP was a novel target of miRNA-29c. We found that miRNA-29c directly interacted to the 3′-UTR of TTP and repressed TTP expression. Consistent with this notion, an inverse correlation between miRNA-29c and TTP expression was detected in podocytes.

In summary, we demonstrated that miRNA-29c is a component of high glucose-induced inflammatory response and that miRNA-29c promoted the progression of DN by targeting TTP. These findings provided new insights into the role of miRNA-29c in the diabetic milieu and would open a new avenue for a therapeutic intervention of DN.

## Material and Methods

This study, including all the tissue sample collection and experiment protocols, was approved by the Ethical Committee of the First Affiliated Hospital of Zhengzhou University. Besides, informed consent was obtained from all study participants prior to sample collection. In addition, all methods and protocols were performed under the guidelines and regulations of Ethical Committee of the First Affiliated Hospital of Zhengzhou University.

### Specimens from DM Patients and Controls

The blood and urine samples were obtained from 100 subjects who attended the First Affiliated Hospital of Zhengzhou University between October 2014 and July 2015, including 32 patients with DN, from whom 10 renal biopsy samples were collected, 33 patients with type 2 DM (T2DM), and 35 normal healthy subjects who at-tended the hospital for routine physical examination and were confirmed to be normotensive and without any chronic diseases, such as DM, kidney diseases or other serious diseases. Additionally, for the comparison in pathological analysis, 10 normal renal tissue samples were collected 5 cm away from the tumor of patients with renal carcinoma undergoing surgical treatment.

### miRNA Microarray Analysis

Pooled plasma and urine sediment samples of eight DN patients and eight age- and sex-matched health control subjects and three paired renal tissues from patients with DN and normal controls were used in the analyses of miRNA expression with miRCURY LNATM miRNA Array chip technology (Exiqon, Vedbaek, Denmark) to detect the differences in the expression of miRNAs and search for miRNAs that might regulate TTP mRNA, using combined database analyses such as miRbase (http://www.mirbase.org) and TargetScan Human (http://www.targetscan.org/vert_71). The miRbase database provided information on all the published miRNA sequences and annotation, and the TargetScan Human could predict biological targets of miRNAs.

The full-length mRNA of mouse TTP (NM_011756) was obtained from the National Center for Biotechnology Information (NCBI) database. The miRNA-29c sequence was obtained from miRNA sequence database (miRbase). TargetScan was used to assess potential targets sites for miRNA-29c.

### Cell Lines and Cell Culture

Conditionally immortalized mouse podocytes (MPC5) were kindly provided by Prof. Peter Mundel (Mount Sinai Medical School, New York, NY, USA) and cultured as reported previously^22^. Briefly, the podocytes were seeded onto BD BioCoat Collagen I plates (BD Biosciences, Franklin Lakes, NJ, USA) and cultured in RPMI 1640 medium (Gibco, Gaithersburg, MD, USA) at 33 °C in the presence of 10 units/mL of mouse recombinant IFN-γ (Invitrogen, Carlsbad, CA, USA) to enhance the expression of a thermosensitive T antigen. To induce differentiation, the podocytes were maintained in RPMI 1640 medium (Gibco) at 37 °C without IFN-γ for 10–14 days.

### Quantitative RT-PCR

Total RNA was extracted using TRIzol reagent (Invitrogen) following the manufacturer’s instructions. To measure miRNA-29c expression, total RNA was reverse-transcribed using the Two Step Stemaim-it miR qRT-PCR Quantitation Kit (Shanghai Novland Co., Ltd, Shanghai, China). To measure the mRNA expression, total RNA was reverse-transcribed using the HiScript Q RT SuperMix kit for qPCR (Nanjing Vazyme Biotech Co. Ltd, Nanjing, China). Quantitative PCR reactions were performed on the SLAN Real-Time PCR system (Shanghai Hongshi Medical Technology Co. Ltd, Shanghai, China) using Two Step Stemaim-it miR qRT-PCR Quanti-tation Kit (Shanghai Novland Co., Ltd, Shanghai, China) and AceQTM qPCR SYBR Green Master Mix (Nanjing Vazyme Biotech), respectively. The U6snRNA or GAPDH were used as normalization controls and the relative expression levels were calculated using the 2^−ΔΔCt^ method^42^. Gene-specific primers used in the present study are listed in Table 3.

**Table 3:** The primers used for real-time PCR.

Target gene	Forward sequence (5′ to 3′)	Reverse sequence (5′ to 3′)
TTP	CTTTCCCCTTCTGCCTTCTC	TGGTGCTGGGGGTAGTAGAC
IL-6	CCGGAGAGGAGACTTCACAG	CAGAATTGCCATTGCACAAC
TNF-α	GCTGAGCTCAAACCCTGGTA	CGGACTCCGCAAAGTCTAAG
GAPDH	AGAACATCATCCCTGCATCC	CACATTGGGGGTAGGAACAC
miRNA-29c	CTGACCTTAGCACCATTTGAAATC	TATCGTTGTACTCCACTCCTTGAC
U6	ATTGGAACGATACAGAGAAGATT	GGAACGCTTCACGAATTTG

Total RNAs from renal tissues, plasma, and urine sediments were isolated using the TRIzol reagent (Invitrogen). cDNA was synthesized using reverse transcriptase. All PCR reactions were run using a certified PCR instrument (Thermo Fisher Scientific, Waltham, MA, USA), according to the manufacturer’s instructions. The SLAN Real-Time PCR system used in the present study was obtained from Shanghai Hongshi Medical Technology Co. Ltd (Shanghai, China).

### Gain-of-Function and Loss-of-Function Assays for miRNA-29c

The *in vitro* gain-of-function assay with miRNA-29c mimics and the loss-of-function assay with miRNA-29c inhibitor were carried out using cell transfection techniques. All these oligonucleotides were synthesized by RiboBio (Guangzhou RiboBio Co., Ltd, Guangzhou, China). The oligonucleotide sequences of miRNA-29c mimics, inhibitor, and their corresponding controls were as follows: miRNA-29c mimics: Sense: 5′-UAGCACCAUUUGAAAUCGGUUA-3′, and Anti-sense: 5′-UAACCGAUUUCAAAUGGUGCUA-3′; miRNA-29c mimics negative control: Sense: 5′-UCACAACCUCCUAGAAAGAGUAGA-3′, and Anti-sense: 5′-UCUACUCUUUCUAGGAGGUUGUGA-3′; miRNA-29c inhibitor: 5′-UAACCGAUUUCAAAUGGUGCUA-3′, and miRNA-29c inhibitor negative con-trol: 5′- UCUACUCUUUCUAGGAGGUUGUGA-3′.

The mouse podocytes were cultured to about 80% confluence in 6-well plates and were transfected with 30 nM of mimics or 100 nM of inhibitor in the presence of Lipofectamine 2000 (Invitrogen), according to the manufacturer’s instructions. At 6–48 h after transfection, the cells were harvested for further experiments.

### Luciferase Reporter Assay

The fragment of the TTP wild-type (Wt) 3′-UTRs, including the miRNA-29c binding site within the 3′-UTRs, was amplified by PCR from mouse podocyte genomic DNA. The PCR primers for TTP were as follows: TTP-SacI-UTR1, 5′-GAAGAGCTCCAAGTGCCTACCTACCCAGTATGGA-3′ (forward), and TTP-XhoI–UTR2, 5′-GAACTCGAGACTGTCAACTGTCTCCCTCAAACAT-3′ (reverse).

The TTP mutant-type (Mut) 3′-UTR fragment containing mutants on the miR-NA-29c binding site was amplified from the 3′-UTR fragment using the following primers: TTP-SacI-UTR1 + TTP-mut-UTR4 (5′-CCCAGCCCTCGTGGTGGGAAATGGGTTATTGCATCTTGGAATGTC-3′) and TTP-mut-UTR3 (5′-AACCCATTTCCCACCACGAGGGCTGGGGCAGGTCCCTAGTTTGCA-3′) + TTP-XhoI-UTR2 (Underlined bases show the mutation site).

The 258-bp Wt or Mut 3′-UTR was cloned downstream of the pmirGLO vector (Promega, Madison, WI, USA). Firefly luciferase was used as a reporter gene and Renillaluciferase as a normalized control. Then, the mice podocytes were co-transfected with the Wt or Mut TTP 3′-UTR reporter vector and miRNA-29c mimics or miR-control using ECM2001electrophoretic transfer (BTX Technologies, Inc., Hawthorne, NY, USA). After 36-h transfection, the cells were harvested and luciferase activity was determined using the Dual-Luciferase Reporter Assay System (Promega) on a Centro XS3 LB 960 Microplate Luminometer (Berthold, Germany), according to the manufacturer’s instructions.

### Western Blotting Analysis

For preparation of total cellular proteins, the treated and control cells were washed with PBS and then lysed with RIPA buffer (50 mmol/L Tris-base, 150 mmol/L of NaCl, 0.1% SDS, 1% Triton X-100, 0.5% sodium deoxycholate, 1 mmol/L of sodium orthovanadate, 10 mmol/L of sodium fluoride, 1% protease inhibitor cocktail) for 15–20 min on ice. After centrifugation at 13,400 g for 15 min, the supernatants were collected and the protein concentration was quantified by the BCA protein as-say (Sigma-Aldrich Corporation, Saint Louis, MO, USA). The samples with equal amounts of 10 µg of total proteins were prepared in SDS-PAGE loading buffer, heated at 100 °C for 5 min, separated on 10% polyacrylamide gel, and then transferred to PVDF membranes (Roche, Indianapolis, IN, USA). The membranes were blocked in 5% non-fat milk in TBST buffer (Tris Buffer Saline containing 0.1% Tween-20) for 2 h at room temperature, and subsequently incubated with rabbit monoclonal anti-mouse TTP antibody (diluted 1:500) (Santa Cruz Biotechnology, Inc., Dallas, TX, USA) at 4 °C overnight. After extensive washing with TBST buffer, the blots were incubated with HRP-conjugated secondary antibody for goat anti-rabbit IgG (diluted 1:1000) (Abcam PLC, Cambridge Cambridgeshire, UK) for 2 h at room temperature. After extensive washing with TBST buffer, the target proteins were detected by enhanced chemoiluminescence reagents (ECL).

### ELISA

The detection of IL-6 and TNF-α was accomplished using an IL-6 or TNF-α ELISA kit (Elascience, Wuhan, China). The podocytes were cultured in 25-cm^2^ flasks with medium containing 10% FBS for 24–48 h and transferred to serum-free medium before initiation of experiment. At the end of 48 h, the cell-free culture medium was collected and analyzed using the ELISA Kit according to the manufacturer’s instructions. The optical density at 450 nm was determined using aniMark Microplate Reader (Bio-Rad Laboratories Inc., Berkeley, CA, USA).

The blood and urine samples were centrifuged at 1,500 g for 10 min and the supernatants were collected and stored at −80 °C until analysis. The levels of human IL-6 and TNF-α in plasma and urinary supernatants were measured using ELISA kits (Shanghai ExCell Biology, Inc., Shanghai, China).

Creatinine (Cr), blood urea nitrogen (BUN), hemoglobin A1c (HbA1c), triglyceride (TG), and albumin were measured using an automatic biochemical analyzer (CS400) (Changchun Dirui Medical Technology Co Ltd, Changchun, China).

### Statistical Analysis

All the experiments were repeated for at least three times. The data were ex-pressed as means ± SEM. Statistical analyses were conducted with SPSS 17.0 software, using the unpaired Student’s t-test for comparisons of two groups or one-way ANOVA for multiple group comparisons. Spearman’s rank correlation coefficient was adopted to analyze the correlation between parameters. *P* < 0.05 was considered statistically significant.
