# Author Correction: MiRNA-29c regulates the expression of inflammatory cytokines in diabetic nephropathy by targeting tristetraprolin

**DOI:** 10.1038/s41598-018-25612-4

**Published:** 2018-05-02

**Authors:** Jia Guo, Jing Li, Jing Zhao, Shuguang Yang, Luyao Wang, Genyang Cheng, Dong Liu, Jing Xiao, Zhangsuo Liu, Zhanzheng Zhao

**Affiliations:** 1 Nephrology Hospital, The First Affiliated Hospital of Zhengzhou University, NO. 1 Jianshe Eastern Road, 6th Floor of NO. 7 Building, Erqi District, Zhengzhou, 450052 China; 2 Zhengzhou University Institute of Nephrology, Zhengzhou, 450052 China; 3 Institute of Clinical Medicine, The First Affiliated Hospital of Zhengzhou University, Zhengzhou, 450052 China

Correction to: *Scientific Reports* 10.1038/s41598-017-01027-5, published online 24 May 2017

This Article contains errors. Figure 6B was misassembled during the preparation of the manuscript: an incorrect image was used for the panel illustrating GADPH loading controls. The corrected Figure 6B appears below as Figure 1.

**Figure 1:**
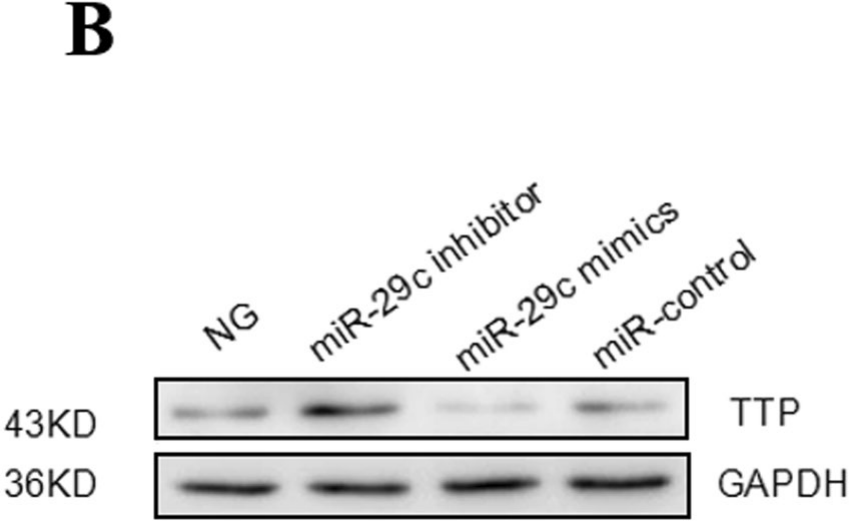
miRNA-29c targets TTP. (**A**) The expression of miRNA-29c in podocytes transfected with mimics (30 nM) or inhibitors (100 nM) for 6 h. (**B**) Western blot analysis showed the expression of TTP in podocytes transfected with mimics or inhibitors for 36 h. (**C**) The quantified Western blot data shown in graph format. (**D**) qRT-PCR analysis of TTP mRNA expression in podocytes transfected with mimics or inhibitors. (**E**) Podocytes were transfected with miRNA-29c inhibitor (100 nM) and treated with HG (25 mM) for 36 h as compared with NG. (**F**) The quantified Western blot data shown in graph format. (**G**) qRT-PCR analysis of TTP mRNA expression in podocytes transfected with miRNA-29c inhibitors (100 nM) and treated with HG (25 mM) for 24 h. Results were obtained from three independent experiments. The data are shown as mean ± SEM. *P < 0.05 compared with control. #P < 0.05 compared with high glucose.

The conclusions of the article are unaffected by this correction. The Authors apologize for the error and any inconvenience caused.

